# Client-Focused Security Assessment of mHealth Apps and Recommended Practices to Prevent or Mitigate Transport Security Issues

**DOI:** 10.2196/mhealth.7791

**Published:** 2017-10-18

**Authors:** Jannis Müthing, Thomas Jäschke, Christoph M Friedrich

**Affiliations:** ^1^ Department of Computer Science University of Applied Sciences and Arts Dortmund Dortmund Germany; ^2^ Department of Business Information Systems FOM University of Applied Sciences Essen Germany

**Keywords:** mobile health, mobile apps, data security, computer security, confidentiality, health information technology

## Abstract

**Background:**

Mobile health (mHealth) apps show a growing importance for patients and health care professionals. Apps in this category are diverse. Some display important information (ie, drug interactions), whereas others help patients to keep track of their health. However, insufficient transport security can lead to confidentiality issues for patients and medical professionals, as well as safety issues regarding data integrity. mHealth apps should therefore deploy intensified vigilance to protect their data and integrity. This paper analyzes the state of security in mHealth apps.

**Objective:**

The objectives of this study were as follows: (1) identification of relevant transport issues in mHealth apps, (2) development of a platform for test purposes, and (3) recommendation of practices to mitigate them.

**Methods:**

Security characteristics relevant to the transport security of mHealth apps were assessed, presented, and discussed. These characteristics were used in the development of a prototypical platform facilitating streamlined tests of apps. For the tests, six lists of the 10 most downloaded free apps from three countries and two stores were selected. As some apps were part of these top 10 lists in more than one country, 53 unique apps were tested.

**Results:**

Out of the 53 apps tested from three European App Stores for Android and iOS, 21/53 (40%) showed critical results. All 21 apps failed to guarantee the integrity of data displayed. A total of 18 apps leaked private data or were observable in a way that compromised confidentiality between apps and their servers; 17 apps used unprotected connections; and two apps failed to validate certificates correctly. None of the apps tested utilized certificate pinning. Many apps employed analytics or ad providers, undermining user privacy.

**Conclusions:**

The tests show that many mHealth apps do not apply sufficient transport security measures. The most common security issue was the use of any kind of unprotected connection. Some apps used secure connections only for selected tasks, leaving all other traffic vulnerable.

## Introduction

### Mobile Health Apps

With the emergence of smartphones, ubiquitous Internet access and the app ecosystems around, health information technology also found its way to these devices. Mobile health (mHealth) describes using mobile devices to facilitate medical or health-related purposes [1]. Among many other apps, mHealth apps may offer a means of communication between patients and medical professionals. They also give patients the ability to keep track of their medical characteristics [2-5].

In developing countries, smartphones are often the only means of Internet access. mHealth apps on smartphones can thus help to minimize discrepancies in health care worldwide [6,7]. Because they are used in a diverse set of medical apps, they have a heightened need for protection [8]. To offer any security, device vendors must ensure fast security patches for smartphones. This represents an issue especially for low-cost Android-based devices [9]. Beyond device security, security of data in transport is relevant and will be the focus of this paper.

European privacy regulations set an additional baseline for data handling by app providers [10]. The regulations are binding in European countries only. The Privacy Code of Conduct on mHealth apps by the European Commission represents an important initiative outlining the heightened security requirements for mHealth apps [11].

Studies have shown that there is an existing concern about information security [12,13]. mHealth-related apps that do not provide appropriate security might impede the growth of the sector.

### Transport Security

To provide information or to enable the transmission of (medical) data to a service provider, an app must communicate with servers. As soon as data are sent through public infrastructure, data can potentially be observed, modified, or redirected. Without any protection, this endangers the integrity of data displayed, gives away potentially sensitive data, and enables malicious parties to impersonate the victim.

The transport layer security (TLS) protocol makes up the foundation of the modern Internet’s security infrastructure. It was designed to give protection against the aforementioned problems, offering authentication, data integrity, and confidentiality through asymmetric and symmetric cryptography. In the recent past, protocol weaknesses such as Padding Oracle On Downgraded Legacy Encryption [14], Browser Exploit Against SSL and TLS, Factoring RSA Export Keys, and others, as well as implementation problems such as Heartbleed [15,16] and Apple’s goto fail bug [17] have arisen. The use of older protocol versions or deprecated implementations can lead to these or other issues surfacing and compromising the security and privacy of users.

Some prior research examined app source code for transport security issues using static code analysis [18-22], showing clearly that many apps are not using aforementioned up-to-date security measurements and consequently putting users at risk. The methods used in this paper will rely on the observation of communication between the client app and servers, and thus enabling observations under real-world conditions. Consequently, the research presented also does not focus on the analysis of data locally stored on a smartphone [23]. Other transport security issues relevant to this research are listed as part of the Open Web Application Security Project Mobile Top 10 [24].

Apps on mobile devices conceal details of communication with their servers from end users. Whereas a user of a website might be able to identify a website as insecure and be warned about certificate issues, a mobile app does not automatically warn the user about invalid certificates or missing encryption [25]. This highlights the importance of independent evaluation of mobile app transport security.

### Prior Work

In existing research, metadata of mHealth apps on iOS and Android app stores were analyzed and evaluated [26]. No test or technical analysis was performed in that publication. Other research focused on health-related apps in Chinese App Stores [27]. This paper also did a comprehensive metadata analysis and a manual screening of popular Chinese mobile apps [27]. The security analysis is limited to viewing of documentation or auditing report availability from the app’s developer. The paper did find that information security was absent in 97% of the evaluated apps [27].

Furthermore, a framework for risk assessment of mHealth apps was proposed [8]. The research focuses on evaluation and categorization criteria for apps and represents an excellent motivation for this work.

In other existing literature, a study on security aspects of Android apps was performed, taking an in-depth look at 22 mHealth apps [28]. Here data in transit as well as device data (on Secure Digital cards or in system log files) were considered to evaluate the apps. Their results contain the finding that 18 of these apps send data unencrypted over the Internet.

Beyond the field of health-related security analysis, Gagnon et al proposed the AndroSSL Platform to test Android apps regarding transport security [29]. The approach presented here was to test apps in an Android virtual device, utilizing a virtual test bed for Android apps [30]. This enabled to record a test once and repeat it multiple times automatically. The focus was on certificate validation when secure connections were used. By being able to repeat a test automatically, it was possible to issue different secure sockets layer (SSL) certificates to find out whether the client validated them correctly. These or similar test scenarios are also found in other relevant research [22,31]. This led to the incorporation of similar tests into the research presented in this paper.

The primary objective of the research presented in this paper was to assess prominent transport security issues in popular mHealth apps and to outline ways for developers of such apps to mitigate these issues.

The following Methods section will first outline the app selection criteria. A description of all the aspects analyzed during the tests will be given in this section. Subsequently, the system used for the tests will be described. In the Results section, the apps selected for testing by the criteria described before will be given, followed by a description of how the previously described system was applied for testing. The last section discusses common security concerns found during the tests, compares prior work with this paper, and recommends practices to mitigate the security issues discussed.

## Methods

### App Selection

To achieve appropriate diversity in the test pool, mHealth apps from different European countries were chosen. To mitigate any platform-dependent bias, apps for Android as well as for iOS were tested.

### Relevant Transport Security Considerations

This section will describe each characteristic that will be considered in the tests performed later in this paper.

**Figure 1:**
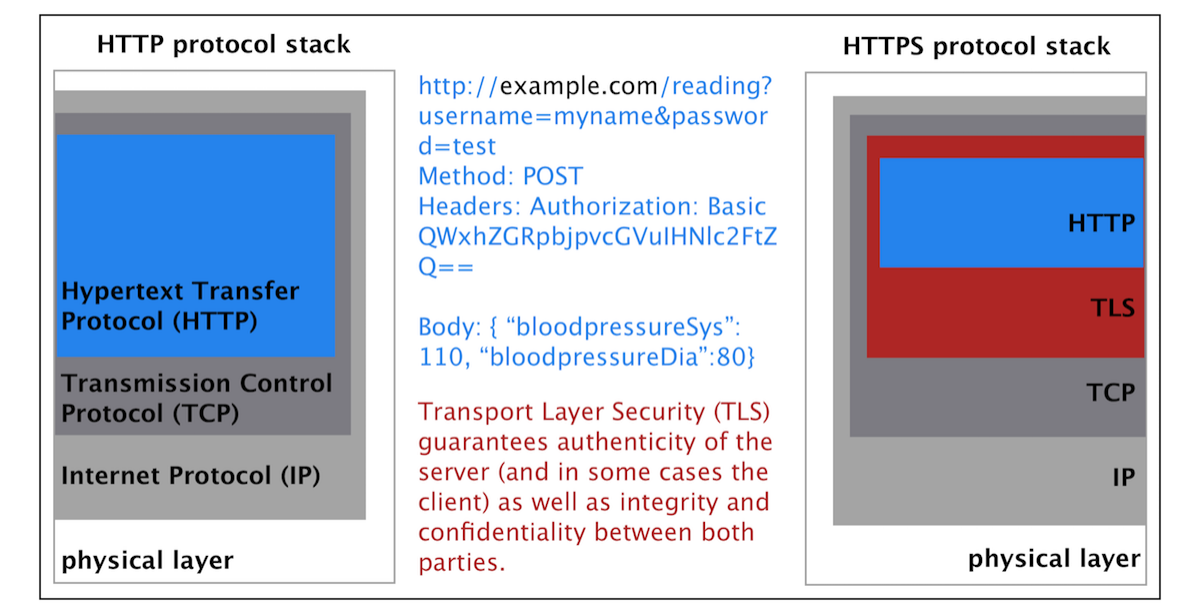
The Hypertext Transfer Protocol (HTTP) and Hypertext Transfer Protocol Secure (HTTPS) protocol stacks. The topmost layers (transport layer security [TLS] and HTTP itself) are of most interest. The HTTP protocol contains any relevant data sent to or received from the server. Examples for HTTP data are written in blue. These data are readable by any third party when TLS is not used. When HTTP is used on top of TLS, these data are encrypted. Additionally, TLS ensures the integrity of the messages exchanged and the authenticity of the server and in some cases the clients.

HTTP (Hypertext Transfer Protocol) is widely used by mobile apps to facilitate server-client communication [32]. This paper focuses on information transmitted utilizing this protocol. HTTP is an application layer protocol (layer 4 in the Transmission Control Protocol and Internet Protocol stack) and can be used on top of a secure TLS connection [33,34]. TLS and its predecessor SSL are designed to ensure confidentiality (encryption), integrity, and authenticity between the parties involved in the communication. The protocol utilizes asymmetric cryptography and a public key infrastructure during its initial handshake and key exchange. Later communication is symmetrically encrypted [35,36]. In Figure 1, the protocol stacks for unprotected HTTP and protected HTTPS (Hypertext Transfer Protocol Secure) are illustrated. The version of the transport security protocol in use is of high relevance to the security of a connection. Earlier versions of TLS and SSL had severe security issues [14,37,38]. This makes testing for the use of HTTPS in general and for the TLS version imperative.

By default, a TLS implementation, for example, in a browser or in a mobile operating system trusts a number of root public certificates from certificate authorities [39]. When an app makes a secure connection to a server, this server authenticates itself with a certificate. The TLS client on the smartphone validates that this certificate was derived from one of its trusted certificates. Because these lists of trusted certificates are not controllable by the app developer, it is possible that it contains compromised certificates. As soon as an app trusts such a rogue certificate, the owner of the rogue certificate can issue valid certificates for any domain visited by the device and can, therefore, pretend to be any server [21]. This enables an attacker to act as a middle man (man-in-the-middle [MitM]) between the client and the server, leading to undermined integrity of server responses and loss of privacy between the client (and thus the user of an app) and the server [40]. In Android version <7, the user can install such a certificate himself. In later versions, a user cannot install additional CA (Certificate Authority) certificates [41]. It should be noted that system integrity is required for the validation of certificate trust chains to work. Android’s inconsistent history with system security in the past could make it more likely that an attacker might use unpatched issues to gain privileges on the system and install any certificates wanted or to do further harm [42,43]. A major issue with Android phones is the lack of willingness in phone manufacturers to ship security updates to their adoptions of Android, leading to a high degree of version fragmentation in the market [9,44]. iOS gives the user a way to install a trusted CA certificate manually.

To make sure an app only connects to the correct servers, apps can be shipped including several trusted certificates. When a secure connection is made, the app validates the server certificate against these certificates. As the app bundle is signed by the developer and consecutively by the store operators (Apple and Google, respectively), it cannot be tampered with later [45,46]. This technique is called pinning. Whereas it brings some important advantages, shipped trusted certificates can expire, making the app unable to connect to its servers. It is also possible that the necessity arises for a certificate to be revoked. This will require the app to be repackaged. Pinning is similar to HTTP public key pinning (HPKP) but does not require changes to the server [47].

Because HPKP depends on a server configuration, it may not prevent all MitM attacks [48]. During the tests, a self-issued CA certificate is utilized and installed on devices used for testing to inspect encrypted traffic in a part of the tests. When a connection attempt is consistently aborted by the client while the proxy is presenting a certificate derived from the aforementioned CA certificate, pinning is likely to be used by the app. Pinning is enabled if no connections to an app’s backend can be made through the proxy.

Another set of tests is inspired by Gagnon et al. It consists of several scenarios to test the certificate validation of TLS implementations in apps [29]. Four of these are part of the tests performed in this paper. In every scenario, the proxy serves a different TLS certificate for each domain requested. The certificates are all invalid and should be rejected by the client app under test. These are the characteristics of the certificates served in the scenarios:

1. Correct domain name, signed by an untrusted CA certificate
2. Self-signed certificate for the domain requested
3. Static host name, signed by a trusted CA
4. Self-signed for a static hostname

Because each scenario requires a separate manual test, only these four scenarios were selected [29]. The last scenario in Gagnon et al’s paper did not yield any further results and was therefore excluded from the setup.

Next, the leakage of information is considered. Cookies are used by servers to hold session information [49]. They are transmitted as HTTP header fields. If it is possible to reuse an intercepted cookie, the interceptor can hijack a session. Leaked cookies can also reveal user data directly [50]. Cookies should be protected by a secure connection. A secure cookie scheme can also mitigate the issues [51].

Cookies are one way to identify a client to the server. Users can be authenticated by all kinds of tokens or parts of an HTTP request. Therefore, the system to be developed will look for cookie, set-cookie, and authorization headers.

The authorization header field can contain one of multiple possible values of interest. It may leak usernames and passwords [52], OAuth2 Bearer tokens [53], or other sensitive information.

Additionally, the body and URL string of each request and response will be evaluated for any username or password leaks.

Lastly, the server location is relevant, as it has consequences for the jurisdiction applied. As mentioned earlier, servers outside Europe are not under the European privacy regulation.

### Development of System for Semiautomatic Tests of Relevant Transport Security Issues

To be able to rapidly and thoroughly test for the issues discussed above, a Web-based app was developed. This Web-based app should enable users to test apps for vulnerabilities while also facilitating more in-depth analysis. The software is called BProxy.

The app was based on the Zed Attack proxy and was started as a fork of version 2.4.3 [54]. The main points of reusing the existing code were the proxy inspection and dynamic certificate–issuing codebase. Changes were made to dynamically modify how certificates for requested domains are issued (to enable the certificate validation scenarios discussed earlier). A representational state transfer (REST) application programming interface was designed to expose automatic creation and control of proxies [55]. Additionally, an HTTP server exposes the Angular2-based user interface. This Web-based app interacts with the REST interface to control the proxy.

The architecture of BProxy was engineered with fast and simple extensibility in mind. Each single transport security consideration was tested by a separated module. Modules can implement interfaces to register for callbacks and influence properties of TLS handshakes (for the TLS certificate validation tests). Multimedia Appendix 1 shows the general software architecture of the tool. Additionally, BProxy has been released as open source software to help reproducibility of the research presented [56].

During each test of an app, the proxy works in sessions. Before the start of each session, the app under test is relaunched. During a session, the user interacts with it. Any registration or log-in actions are repeated.

First, this enables the system to separate domains used by the app from other domains the device might communicate with (background tasks, changing ads displayed in the app). A domain present in more sessions is more likely to be connected to the app under testing. Second, some sessions are used for the certificate validation tests described earlier.

**Figure 2:**
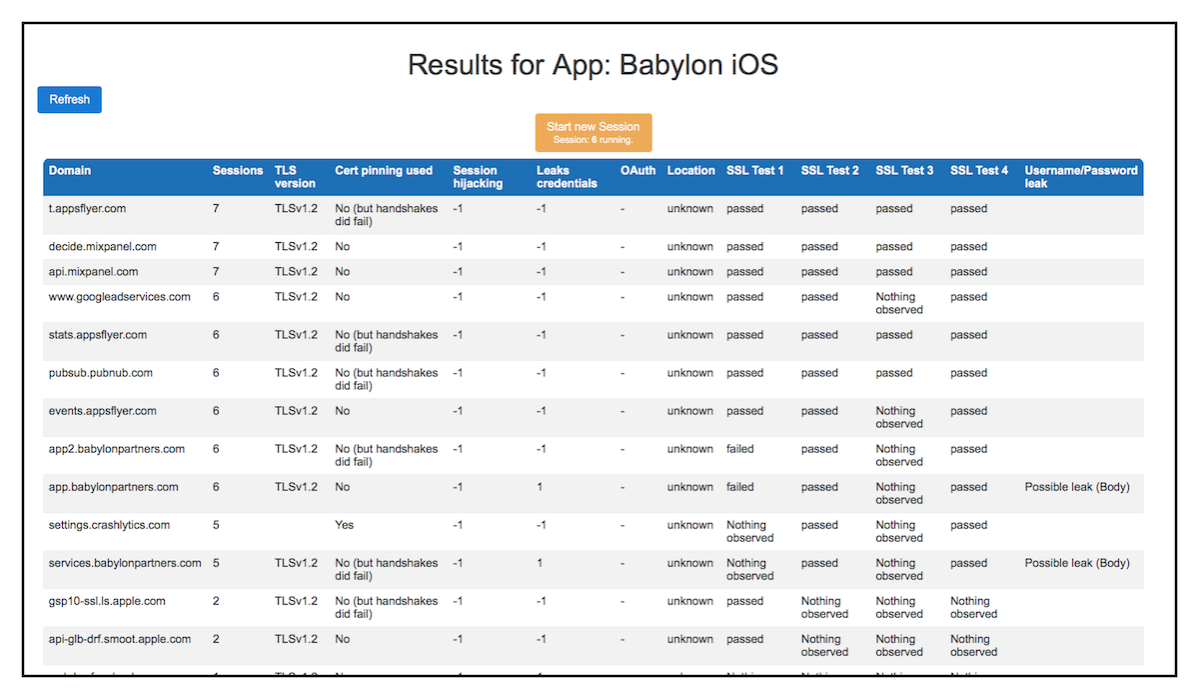
BProxy example results output. The columns inform the user about observations made by the proxy: the Transport Layer Security (TLS) version used (TLS version), whether certificate pinning was used (Cert pinning used), whether cookies were observed (Session hijacking), whether authentication tokens were visible (Leaks credentials), if OpenAuthorization (OAuth) tokens were observed (OAuth), the server location for the domain visited (Location), the results for the certificate validation tests (SSL Test 1-4), if usernames or passwords were observed (Username/Password leak). More Information on BProxy’s output can be found on the Web.

After the necessary number of sessions, a list of domains will be shown. During our tests, two without certificate modifications and four with different certificate validation tests must be run. The results are displayed per domain that the app communicated with. The modules mentioned earlier are responsible for generating these results. Where possible, a user can also display all request and response pairs that are responsible for a certain result displayed. This enables validation of the automatically generated results and further in-depth analysis. The source code for BProxy is available on the Web [56]. An example of how it presents its results is shown in Figure 2.

### Limitations

The platform developed as part of the research for this paper should enable even less technology-affine users of mobile apps to conduct tests and get results. These results should give an indication of the value the app’s developer assigns to security. As a direct result of the intention of developing such a tool, the choice was made early on to develop it as a Web-based platform. This choice brought certain design limitations. First, the analysis is based on the use of a proxy running on a unique port assigned to the app under test. This proxy can simply be configured on user's devices. It is possible for an app to ignore system proxy setting on Android and iOS, but during all tests, no apps ignored the proxy and any traffic was apparently observable.

As described, the developed system works only semiautomatically. This is to enable tests on apps from the respective app stores on Android as well as on iOS. No research on data locally stored on mobile devices was performed.

## Results

### App Selection

Apps are selected by popularity in a relevant category from the Apple App Store as well as from Google Play Store. As mHealth apps are tested, the *medical* category is the most relevant. To diversify the test pool as much as possible, lists of most downloaded, free apps from three countries are considered. European privacy regulations are part of the considerations in this paper, therefore Germany, France and the United Kingdom—the most populated countries in Europe—were selected. The top 10 lists have been retrieved from App Annie on January 10, 2017 [57]. Top lists for a specific day are available after registration on the website. Whereas more exact app descriptions and results are available in the Multimedia Appendices 2 and 3, the apps were further categorized for better understanding of the test results. The categories of the apps tested are summarized in Table 1. The categories for each of the apps are part of the app descriptions in Multimedia Appendix 3.

### Performing the Test Using the BProxy Tool

The test results were obtained utilizing the BProxy tool. The tool displayed results on a per domain basis. The first step in analyzing the output of the tool is the filtering of domains belonging to the app. These domains appear on top of BProxy’s output, as they are communicated more frequently. The second step is to differentiate between connections to servers belonging to an app and those belonging to analytics or advertising providers. Next, the results in the columns are considered. They can be interpreted directly and contribute to the results presented here. To be able to make assessments regarding the integrity of data displayed by an app and confidentiality between an app and its servers, BProxy displays all request and response pairs for every domain. Requests and responses with app servers are examined and evaluated regarding their impact on integrity and confidentiality. In some cases, further testing, such as modification of server responses to validate integrity concerns, was performed using the Charles Web Debugging Proxy Application [58].

### Summarized Results

Detailed results can be found in the form of two tables for Android and iOS apps in Multimedia Appendix 2. These tables list the results for each characteristic separately for every app. Further details on the apps (developers, top 10 list positions, and short descriptions) can be found in Multimedia Appendix 3.

All tests have been performed between January 17, 2017 and January 27, 2017. The most recent versions of the apps have been downloaded from the respective stores shortly before testing. Table 2 shows the summarized results of our tests. As none of the tested apps facilitated certificate pinning, the row was therefore omitted from the table.

The table shows that there are slightly more security issues in apps on the iOS platform in our test pool.

**Table 1:** Assigned categories of the tested apps.

Assigned category	Android, n (N=25)	iOS, n (N=28)	Total, n (N=53)
Pregnancy or fertility related	8	13	21
Drug information	2	1	3
Reference or learning	5	3	8
Consulting or communication	5	6	11
Health and fitness	3	3	6
Others	2	2	4

**Table 2:** Summarized table of results for Android and iOS apps.

Security issues		Android, n	iOS, n	Total, n
1.	Servers outside European Union countries	7	8	15
2.	No transport layer security for connections	7	12	19
3.	Cookies or secure tokens send over insecure connections	4	7	11
4.	Integrity of content displayed in the app compromised	8	13	21
5.	Username and password sent over insecure connections	1	2	3
6.	Confidentiality between user and app provider compromised	3	5	8
7.	Certificate validation issues present	1	1	2

The most consequential issue observed is the omission of any kind of TLS (No transport layer security for connections) for connections present in 19 apps (36%). Insecure connections can lead to integrity (Integrity of content displayed in the app compromised) and confidentiality (Confidentiality between user and app provider compromised) breaches, as well as to exposed cookies, tokens (Cookies or secure tokens send over insecure connections), and user credentials (Username and password sent over insecure connections). The semantic here was that as soon as a single unencrypted connection was used, the app was counted as not using TLS. Although the other issues are considered separately, they are more likely to occur in apps that fail to apply TLS for server connections.

Apps that do use TLS-secured connections were tested regarding their certificate validation mechanism as described. A failure to validate a certificate correctly (Certificate validation issues present) can expose all traffic sent through the TLS-secured connection to be exposed. This renders integrity, confidentiality, and authenticity protections otherwise offered by TLS useless. Two apps (4%) failed to validate server certificates correctly.

A total of 21 apps (40%) failed to protect the integrity of data they display, and a total of 11 apps (21%) failed to protect session data in transport (cookies or tokens), thus enabling attackers to hijack a session. Three mHealth apps (6%) sent user log-in credentials over insecure connections, whereas 8 (15%) compromised confidentiality of communication between the app and its servers.

Additionally, 15 apps (28%) used servers outside the European Union (EU). However, 31 more apps (58%) used analytics or advertising services outside EU countries, bringing the number of apps that communicated with servers outside the EU to 46 (87%).

In the most severe cases, apps transmit data (health data, usernames, and passwords) completely unprotected (ie, *iCare Health Monitor*). Other apps fetch menu structure and update prompt semantics (when and what to display when the app should be updated) through insecure channels. This enables third parties in privileged positions to hijack vulnerable parts of the app. Confidentiality issues were also popular, mostly because of the use of unsecured connections to retrieve content specific to a user's interest or condition. The *Pregnancy+* app, for example, automatically retrieves data through an unprotected (HTTP) connection for the week of the user's pregnancy. This can expose the state of the pregnancy to a third party.

Most popular analytics providers used up-to-date transport security standards. There was no general difference between the security concerns found in iOS and Android apps. However, single apps that exist on both platforms do show different security characteristics. For example, whereas the iOS version of the *Pregnancy+ app* is using a secure connection for log-in and transmission of data, the Android app does not use any kind of transport security. Similarly, the iOS version of the *babylon health online doctor* fails one of the certificate validation tests for one specific domain. This issue does not exist in the Android version of the app.

An issue was discovered in the Android version of the German *Apotheke vor Ort* app. The app does use a secure connection but accepts any (even invalid) certificates from the server. This is very problematic, among other things, because the app offers the possibility to send prescriptions (listing diagnosis, treating practitioner, and other sensitive medical details) to local pharmacies.

Developers of apps with critical test results were informed about the issues found in their apps before publication of this paper. As of March 23, 2017, a total of 5 developers reacted to the information shared with them. Four of the answers received were constructive.

## Discussion

It was found that out of 53 apps tested from the three European App Stores for Android and iOS, 21/53 (40%) showed critical results. Out of these 21 apps, all failed to guarantee the integrity of data displayed. A total of 18 apps leaked private data or were observable in a way that compromised confidentiality between apps and their servers; 17 apps used connections without any protection; and 2 apps failed to validate certificates correctly. None of the apps tested utilized certificate pinning. Many apps employ analytics or ad providers, thereby undermining user privacy.

### Common Security Concerns

The results show the following:

1. Analytics services are almost universally used in the apps under testing. Medical apps often handle sensitive data. Analytics services collect data without consideration of the kind of app using the software. Not only do these providers collect data that should potentially be protected, they also often are located outside EU countries and therefore not bound by EU regulations.
2. Many apps tested still use insecure endpoints or a mix of secure and insecure ones (19/53, 36%). Medical and health-related apps require protection of patient's data (authenticity of the apps communication partner and confidentiality between patient and app) and should display uncorrupted data (integrity). The lack of any kind of connection security results in the most severe security risks for users, patients, and providers of mHealth apps.
3. In this paper, pinning in any form was nonexistent in the tests. Whereas a crash reporting and analytics provider and Apple client software utilized pinning during the iOS tests, none of the apps under test on either platform utilized the technique for all connections to their servers.
4. Certificate validation seems to work fine for most apps that use secure connections (35/37, 95%); this is most likely because higher level programming interfaces are used. There are, however, cases in which apps accepted untrustworthy certificates. In one case, the app *Apotheke vor Ort* accepted all certificates, rendering TLS essentially useless. This is dangerous, as it seems to use a secure connection until certificate-focused tests are performed. In another problematic case, only one test failed for one domain, indicating an implementation error inside a library used by the app *babylon health online doctor*.

### Comparison With Prior Work

The results presented in this paper were gathered by in-depth inspection and evaluation of the network traffic of selected mHealth apps. This differentiates the approach from metadata-based analysis on a fundamental level [26,27]. On a technical level, it is more comparable to Gagnon et al’s AndroSSL analysis [29]. What makes the approach presented here different from that of AndroSSL is that AndroSSL must be locally run, whereas BProxy can be run as a Web service and used by third parties to test their apps. AndroSSL is limited to the Android platform, as it relies on apps running in an emulator. Whereas iOS apps can be tested in a simulator during development, it is not possible to execute iOS binaries from the App Store on the iOS simulator. They are built for the Advanced RISC Machine architecture while the simulator requires them to be built for the Intel x86_64 architecture. The research presented in the AndroSSL publication was not aimed at mHealth-related apps, but only at transport security issues in Android apps.

Lastly, He et al’s approach of in-depth analysis of apps is more manual than the approach presented here, but it does include more characteristics [28]. Although it does include log and storage analysis, the analysis of transport security is limited to detection of completely unencrypted traffic. For example, old TLS versions or certificate validation issues are not considered.

### Recommended Practices for Transport Security

Most serious security issues are a result of missing or inconsistently implemented security measures. The recommended practices that help mitigate the security issues found are as follows:

- Use HTTPS calls exclusively. Be sure to keep the server (and the client) up to date. Enforce the most current TLS version and prevent a fallback to anything older than TLS 1.2 for any connections.
- Pinning is an option. As any certificate will expire, public key pinning or pinning the certificates of a smaller set of CAs can be a workable alternative to pinning to one certificate exclusively.

Up-to-date TLS for every connection from a mobile app prevents most security issues for data during transport. The following points represent suggestions when a full transition to this is unwanted or impossible for some reason:

- Only send usernames and passwords through secure connections.
- Session cookies or authorization tokens should not be sent over insecure connections.
- Loading resources over an insecure connection can leak user activity to an interested third party in a privileged position. Use secure connections to prevent this.

Because this paper focused on European mHealth apps, the location of servers should be kept in mind. Using a server in the EU gives the data on these servers special protection under European privacy regulations [10].

As mHealth apps often handle sensitive patient data, the use of third-party advertising and mobile analytics services should be seriously questioned and, if possible, avoided, or an opt-out option should be offered [11,59]. Analytics providers collect data not only to present it to the app developer but often also to mine information. The same caution should be exercised when considering the use of advertising services. They also enable extensive user tracking and thus pose a confidentiality risk [59]. Most third-party advertising and analytics services are based outside EU borders and legislation.

Some third-party services offer to deliver updates to apps via untrustworthy and unofficial update channels (not through Google’s Play Store or Apple’s App Store). The security implications of the use of these services are far-reaching and potentially open apps up to remote code injection, putting users at risk of confidentiality breaches and invalidating app integrity [60,61]. Third-party frameworks that use this technique should also be avoided.

### Conclusions

The tests show that many mHealth apps do not apply sufficient transport security measures. The most common security issue was the use of any kind of unprotected connection. Some apps used secure connections only for selected tasks, leaving all other traffic vulnerable.

## Appendix

Multimedia Appendix 1 Architectural diagram of the BProxy tool.

Multimedia Appendix 2 Tables containing detailed results of every app under testing.

Multimedia Appendix 3 Tables listing the apps tested, their top list positions, developer, and a short description.

## Abbreviations

CA: certificate authority
EU: European Union
HPKP: HTTP public key pinning
HTTP: Hypertext Transfer Protocol
HTTPS: Hypertext Transfer Protocol Secure
mHealth: mobile health
MitM: man-in-the-middle
REST: representational state transfer
SSL: secure socket layer
TLS: transport layer security
