# Supplementary figures and images for: Client-Focused Security Assessment of mHealth Apps and Recommended Practices to Prevent or Mitigate Transport Security Issues
Source: JMIR Mhealth Uhealth. 2017 Oct 18;5(10):e147. doi: 10.2196/mhealth.7791 (PMC5666225; doi:10.2196/mhealth.7791)

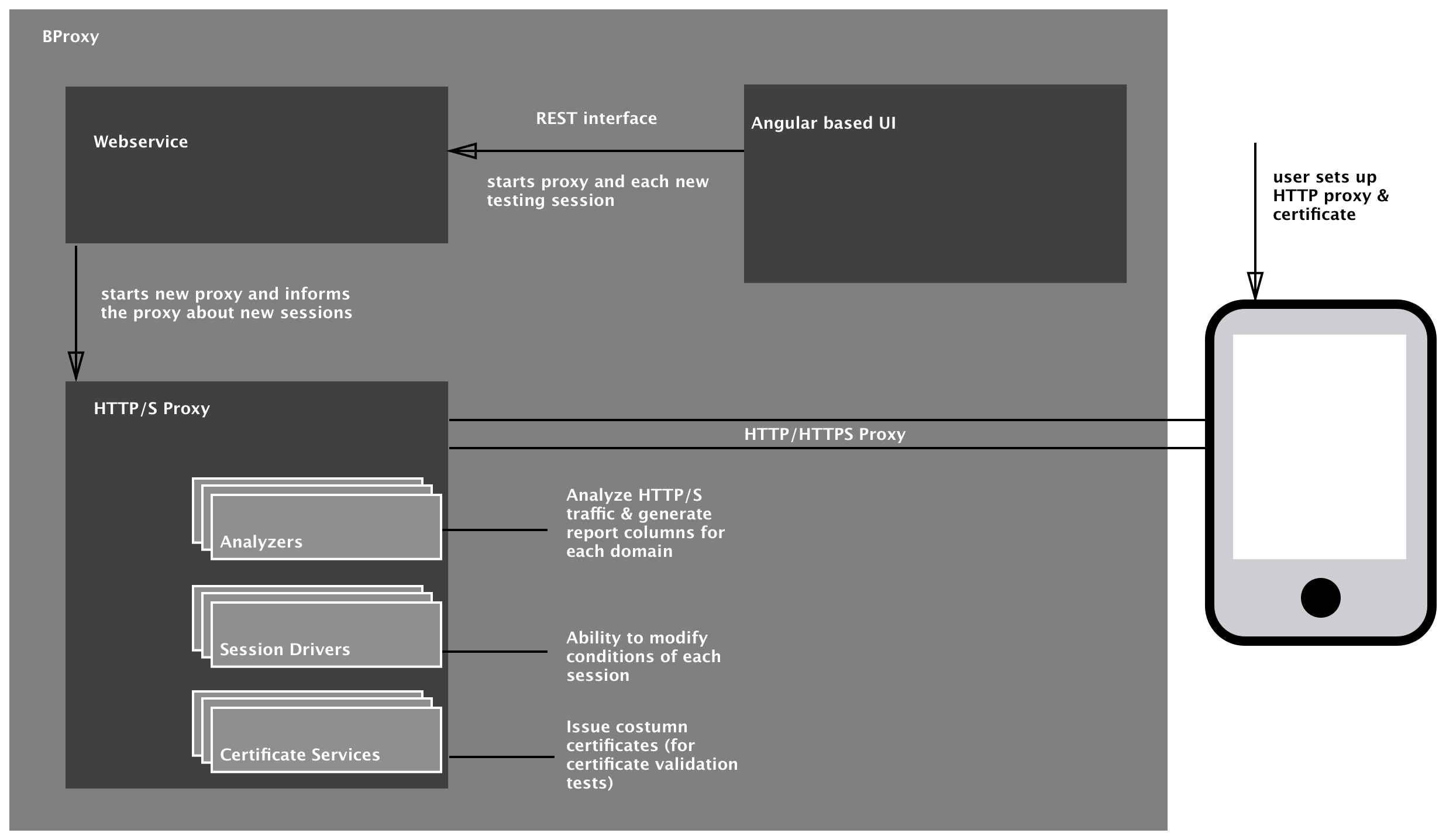

Supplement: Multimedia Appendix 1 [file mhealth_v5i10e147_app1.png]
