# Supplementary material for: Client-Focused Security Assessment of mHealth Apps and Recommended Practices to Prevent or Mitigate Transport Security Issues
Source: JMIR Mhealth Uhealth. 2017 Oct 18;5(10):e147. doi: 10.2196/mhealth.7791 (PMC5666225; doi:10.2196/mhealth.7791)
Supplement: Multimedia Appendix 2 [file mhealth_v5i10e147_app2.pdf]

The following represents the main results of the tests. These are the explanations of the contents of the columns:

Country: This state the country of the top list the app was mentioned on:

Germany (de)
France (fr)
United Kingdom (uk)

EU:

Yes: Server appear to be in the EU only
No: At least one backend server is not inside the EU
No[a]: Ad, tracking or other third party servers used by the app appear to be outside EU countries

TLS Version: States the lowest TLS standard used by a connection. "-" stands for non-TLS secured connections. Distinguishes between analytics servers and servers by the app's backend.

Certificate handling: Contains the tests that failed: these certificates were accepted:

0: Correct domain name, signed by an untrusted CA certificate
1: Self-signed certificate for the domain requested
2: Static host name, signed trusted a CA
3: Self-signed for a static hostname

Session hijacking: cookie or authorization token leaked.

Possible values:
-1: No cookie or authorization token leaked
0: Leaks unlikely because connection secure (pinning used)
1: Connection secured, leaks unlikely
2: Cookies/Tokens exchanged through TLS < 1.2 secured channels
3: Leaks possible, cookies/tokens exchanged through insecure channels (certificate validation broken)
4: Leaks possible, cookies/tokens exchanged through insecure channels (no TLS)

Integrity: Indicates integrity of the information displayed by the app. If there are multiple connections fetching user-facing content, the most critical result is shown.

0: Not possible. Content part of the app or from a secure (pinning) connection
1: Content from a TLS 1.2 secure connection, correct certificate validation
2: Content from a secured connection < TLS 1.2, correct certificate validation
3: Content from a secured connection, incorrect certificate validation
4: Content from an unsecured connection

Username/password handling: Summarizes observations regarding username/email/password handling

0: Nothing sent/not observable (pinning used)
1: Username sent (no pinning, TLS 1.2)
2: Username & password sent (no pinning, TLS 1.2)
3: Username & password sent (TLS < 1.2)
4: Username & password sent (broken certificate validation)
5: Username & password sent (no TLS)

Confidentiality: Summarizes confidentiality concerns found during the analysis of the app.

0: No leak
1: HTTP with TLS < 1.2 calls could reveal user activity

2: Unprotected HTTP (HTTP or certificate validation errors in TLS) calls reveal user activity
3: Transmission of patients/user data through TLS version < 1.2 connections
4: Transmission of patients/user data through a secured connection, incorrect certificate validation
5: Transmission of patients/user data through an unsecured connection

During testing results were given by BProxy on a per-domain/per-request basis. For presentation reasons, the results in this paper are on a per-app basis. Sometimes an app's backend servers and other servers are mentioned separately. In these cases the traffic observed and the domain names led to the conclusions that some servers belong to the app's backend and others to advertising, analytics or other services. It was found that certificate pinning was not utilized by any app tested and the respective column was therefore omitted from the results table.

For better readability, problematic and critical security issues are highlighted.

Android

Country	Name	EU	TLS Version [ad & analytics] / [app servers]	Certificate handling	Session hijacking [ad & analytics] / [app servers]	Integrity	Username, password leak	Confidentiality
Germany								
de, fr, uk	Period Tracker	No[a]	TLS1.2/No connections	ok	1/-	0	-	0
de, fr, uk	Pregnancy+	No	TLS 1.2/-	ok	1/4	4	5	5
de, fr	My Calendar - Period Tracker	No[a]	-/No connections	ok	1/-	0	0	0
de	Apotheke vor Ort	No[a]	-/TLS1.2	0,1,2,3	-1/1	3	0	4
de	Lady Pill Reminder	No[a]	TLS 1.2/No connections	ok	1/-	0	0	0
de	Arznei aktuell	No[a]	TLS 1.2/ TLS 1.2	ok	-1/1	4	2	0
de	AMBOSS Wissen für Mediziner	No[a]	TLS 1.2 / TLS 1.2	ok	1/-1	1	2	0
de	DocCheck Flexikon	No[a]	- / -	-	4/4	4	2	5
de	Blood Pressure Log - MyDiary	-	No connections	-	-	-	-	-
de	Anatomy Quiz	No	- / -	-	1 / -1	0	0	0
France								
fr	Doctolib	No[a]	TLS 1.2 / TLS 1.2	ok	1 / 1	1	0	0
fr	BMI and Weight Loss Tracker	No[a]	TLS 1.2 / No connections	ok	1 / -	0	0	0
fr	L'Appli qui Sauve: Croix Rouge	No	TLS 1.2 / -	ok	1 / 4	4	0	2
fr	Doctisia	No[a]	- / -	-	4 / 4	4	0	2
fr	Ma grossesse Doctissimo	No[a]	- / -	-	4 / -1	4	0	2
fr	Blood Pressure Pro	No[a]	TLS 1.2 / No connections	ok	1 / -	0	0	0
fr	BewellConnect	No[a]	TLS 1.2 / TLS 1.2	ok	- / 1	0	2	0
UK								
uk	citizenAID	-	No connections	-	-	-	-	-
uk	Music & Lyrics for Trolls OST	No	TLS 1.2 / -	ok	1 / -1	4	0	2
uk	Pregnancy Week By Week	No[a]	TLS 1.2 / no connections	ok	1 / -	0	0	0
uk	babylon health online doctor	No[a]	TLS 1.2 / TLS 1.2	ok	-1 / 1	1	2	0
uk	Push Doctor	No	TLS 1.2 / TLS 1.2	ok	1 / 1	1	2	0
uk	NHSGiveBlood	No[a]	TLS 1.2 / TLS 1.2	ok	1 / 1	1	2	0

uk	Ovia Pregnancy & Baby Tracker	No	TLS 1.2 / TLS 1.2	ok	-1 / 1	1	2	0
uk	Ovia Fertility Tracker	No	TLS 1.2 / TLS 1.2	ok	-1 / 1	1	2	0

Table 1 - Android apps test results

iOS

Country	Name	EU	TLS Version [ad & analytics] / [app servers]	Certificate handling	Session hijacking [ad & analytics] / [app servers]	Integrity	Username, password leak	Confidentiality
Germany								
de, fr, uk	Pregancy+	No	- / -	-	4 / 4	4	2	2
de	Pillenalarm	-	no connections	-	-	-	-	-
de	iMamaiPapa	No[a]	TLS 1.2 / TLS 1.2	ok	-1 / 1	1	1	0
de	myPill Birth Control Reminder: Pill, Ring & Patch	No[a]	- / TLS 1.2	ok	-1 / -1	0	1	0
de	PillReminder - Denk an mich	Yes	TLS 1.2 / No connections	ok	-1 / -	0	0	0
de	shop-apotheke	No[a]	TLS 1.2 / -	ok	-1 / 4	4	2	2
de	Arztsuche jameda	No[a]	TLS 1.2 / TLS 1.2	ok	-1 / 1	1	2	0
de	Preventicus Heartbeats – Palpitations unveiled	No	TLS 1.2 / TLS 1.2	ok	-1 / 1	1	0	0
de	Notfallpraxen BW	No	TLS 1.2 / -	ok	-1 / -1	4	0	0
de	DocCheck Flexikon	No[a]	- / -	-	4 / 4	4	2	5
France								
fr	Ma grossesse Doctissimo	No[a]	- / -	-	4 / -1	4	0	2
fr	CitizenDoc	No[a]	TLS 1. 2 / -	ok	-1 / 4	4	0	5
fr	Mon ovulation	No[a]	- / -	-	4 / 4	4	2	2
fr	Parents Grossesse	No[a]	- / -	-	4 / 4	4	2	2
fr	Moi, Bientôt Maman	No[a]	TLS 1.2 / -	ok	-1 / 1	4	2	2
fr	Staying Alive	No[a]	no connections / TLS 1.2	ok	- / 1	1	0	0
fr	VIDAL Mobile	No[a]	TLS 1.2 / -	ok	-1 / 1	4	5	5
fr	iCare Health Mobile	No	- / -	-	1 / -1	4	5	5
fr	Thermo - Suivi de Santé	No[a]	- / no connections	-	4 / -	-	0	0

United Kingdom								
uk	myGP™ Live Life Better	No[a]	TLS 1.2 / TLS 1.2	ok	-1 / 1	1	0	0
uk	citizenAID	-	No connections	-	-	-	-	-
uk	Ovia Fertility Tracker	No	TLS 1.2 / TLS 1.2	ok	-1 / 1	1	2	0
uk	Figure 1 - Medical Cases for Healthcare	No	TLS 1.2 / -	ok	-1 / 1	4	2	0
uk	babylon health online doctor	No[a]	TLS 1.2 / TLS 1.2	0	-1 / 3	3	2	4
uk	Ovia Pregnancy & Baby Tracker	No	TLS 1.2 / TLS 1.2	ok	-1 / 1	1	2	0
uk	My OC	-	no connections	-	-	-	-	-
uk	Ovulation Calculator Fertile Tracker & Calendar OC	No	TLS 1.2 / TLS 1.2	ok	-1 / 1	1	2	0
uk	SystmOnline - patient health management app	Yes	no connections / TLS 1.2	ok	-/ 1	1	2	0

Table 2 - iOS apps test results
