# Supplementary material for: Client-Focused Security Assessment of mHealth Apps and Recommended Practices to Prevent or Mitigate Transport Security Issues
Source: JMIR Mhealth Uhealth. 2017 Oct 18;5(10):e147. doi: 10.2196/mhealth.7791 (PMC5666225; doi:10.2196/mhealth.7791)
Supplement: Multimedia Appendix 3 [file mhealth_v5i10e147_app3.pdf]

Multimedia Appendix 3

Top-lists for the ‘medical’ category of free Android and iOS apps from appannie.com retrieved on January 10, 2017.

Android

#	Country	Name	Publisher	Function	Assigned Category
	Germany				
1.		Period Tracker	Easymobs	Period and fertility tracker	pregnancy/fertility related
2.		Pregnancy+	Health & Parenting Ltd.	Personalized pregnancy information & tracking	pregnancy/fertility related
3.		My Calendar - Period Tracker	SimpleInnovation	Period and fertility tracker	pregnancy/fertility related
4.		Apotheke vor Ort	Wort und Bild Verlag	Drug reservation (e.g. by sending a photo of a prescription), interaction with local drug stores, drug store information, drug information, treatment information, drug interaction checks, special interest news (drugs), information about alternative medical treatment, encyclopedia for medical terms	drug information
5.		Lady Pill Reminder ®	Baviux	Birth control reminder	pregnancy/fertility related
6.		Arznei aktuell	ifap GmbH	Comprehensive drug database, drug interaction information, treatment information etc.	drug information
7.		AMBOSS Wissen für Mediziner	MIAMED GmbH	Medical database, exam preparation for medical topics	reference/learning
8.		DocCheck Flexikon	DocCheck	Public collection of medical data, like Wikipedia, editable by users	reference/learning
9.		Blood Pressure Log - MyDiary	Dr Tomasz Jan Zlamaniec	Enables manual entry of blood pressure, keeps records for later reference	health/fitness

10.		Anatomy Quiz	Alexander Streuer	Learning aid for medical topics	reference/learning
	France				
1.		Doctolib	Doctolib	Making appointments with doctors/health care professionals	consulting/communication
2.		Pregnancy+	Health & Parenting Ltd.	Personalized pregnancy information & tracking	pregnancy/fertility related
3.		My Calendar – Period Tracker	SimpleInnovation	Period and fertility tracker	pregnancy/fertility related
4.		BMI and Weight Loss Tracker	despDev	Weight loss companion app and tracker	health/fitness
5.		L'Appli qui Sauve: Croix Rouge	Croix-Rouge française	First aid tutorials, information, signup for training	reference/learning
6.		Doctisia	vd4soft	Managing health contacts, appointments, displaying a news feed, displaying educational videos	consulting/communication
7.		Period Tracker	Easymobs	Period and fertility tracker	pregnancy/fertility related
8.		Ma grossesse	Lagardère Active Digital	Personalized pregnancy information & tracking	pregnancy/fertility related
9.		Blood Pressure Pro	Jacktindu	Pretends to measure blood pressure from touching the display.	health/fitness
10.		BewellConnect	Visiomed Lab	App to connect to and collect information from several devices	others
	United Kingdom				
1.		citizenAID	citizenAID	Displaying information useful in emergency situations	reference/learning
2.		Pregnancy+	Health & Parenting Ltd.	Personalized pregnancy information & tracking	pregnancy/fertility related
3.		Music & Lyrics for Trolls OST	Henny Sitepu	Incorrectly categorized as medical app:	others

				Displays song lyrics	
4.		Pregnancy Week By Week	Easymobs	Pregnancy tracker app	pregnancy/fertility related
5.		babylon health online doctor	Babylon Health	Offers ability to chat with a server about health concerns, can connect or make appointments with doctors / health care workers	consulting/communication
6.		Push Doctor	Push DR Limited	Offers remote doctor’s consultations	consulting/communication
7.		NHSGiveBlood	NHSBT	Make appointments for blood donations	consulting/communication
8.		Ovia Pregnancy & Baby Tracker	Ovuline, Inc.	Personalized pregnancy information & tracking	pregnancy/fertility related
9.		Ovia Fertility Tracker	Ovuline, Inc.	Period and fertility tracker	pregnancy/fertility related
10.		Period Tracker	Easymobs	Period and fertility tracker	pregnancy/fertility related

iOS

#	Country	Name	Publisher	Function	
	Germany				
1.		Pregancy+	Health & Parenting Ltd.	Personalized pregnancy information & tracking	pregnancy/fertility related
2.		Pillenalarm	Jenapharm GmbH & Co. KG	Birth control reminder	pregnancy/fertility related
5.		iMamaiPapa	GesundBleiben	Personalized pregnancy information & tracking	pregnancy/fertility related
3.		myPill Birth Control Reminder: Pill, Ring & Patch	Bouqt.com Ltd	Birth control reminder	pregnancy/fertility related
4.		PillReminder - Denk an mich	Sanofi	Birth control reminder	pregnancy/fertility related
6.		shop-apotheke	Shop Apotheke B.V	Online drug store	others
7.		Arztsuche jameda	jameda GmbH	Finding doctors near the user and listing ratings, contact details etc.	consulting/communication
8.		Preventicus Heartbeats – Palpitations unveiled	Preventicus GmbH	Heartbeat analyzer utilizing the camera	health/fitness
9.		Notfallpraxen BW	Kassenärztliche Vereinigung	Finding emergency hours for doctor	consulting/communication

			Daden-Württemberg	appointments on weekends or holydays.	
10.		DocCheck Flexikon	DocCheck Medical Services GmbH	Public collection of medical data, like Wikipedia, editable by users	reference/learning
	France				
1.		Pregnancy+	Health & Parenting Ltd.	Personalized pregnancy information & tracking	pregnancy/fertility related
2.		Ma grossesse Doctissimo	Lagardère Active Digital	Personalized pregnancy information & tracking	pregnancy/fertility related
3.		CitizenDoc	CITIZEN DOC	Simple medical consulting, recommends actions and drugs against symptoms	consulting/communication
4.		Mon ovulation	Lagardère Active Digital	Period and fertility tracker	pregnancy/fertility related
5.		Parents Grossesse	UNI-EDITIONS	Personalized pregnancy information & tracking	pregnancy/fertility related
6.		Moi, Bientôt Maman	Betterise Technologies	Personalized pregnancy information & tracking	pregnancy/fertility related
7.		Staying Alive	Association RMC / BFM	crowd sourced defibrillator map	
8.		VIDAL Mobile	VIDAL	Drug & drug interaction information	drug information
9.		iCare Health Mobile	Beijing Jiajia kangkang Co. Ltd.	(Pretends to) Measures health characteristics, guided training, health data visualization	health/fitness
10.		Thermo - Suivi de Santé	Try Sports Now, LLC	body temperature measuring, visualization, health tips,	health/fitness
	United Kingdom				
1.		Pregnancy+	Health & Parenting Ltd.	Personalized pregnancy information & tracking	pregnancy/fertility related
2.		myGP™ Live Life Better	iPlato	Book appointments with a doctor, appointments and medication reminders, track weight and blood pressure	consulting/communication
3.		citizenAID	citizenAID	Displaying information useful in emergency situations	consulting/communication
4.		Ovia Fertility Tracker	Ovuline, Inc.	Period and fertility tracker	pregnancy/fertility related
5.		Figure 1 - Medical Cases for Healthcare	Figure 1, Inc.	Presents itself as an app for medical professionals: database for medical cases, chat with other experts/users.	reference/learning
6.		babylon health online doctor	Babylon Health	Offers ability to chat with a server about health concerns, can connect or make	consulting/communication

				appointments with doctors / health care workers	
7.		Ovia Pregnancy & Baby Tracker	Ovuline, Inc.	Personalized pregnancy information & tracking	pregnancy/fertility related
8.		My OC	Consilient Health Ltd	Birth control reminder	pregnancy/fertility related
9.		Ovulation Calculator Fertile Tracker & Calendar OC	Ovulation Calculator Pty Ltd	Period and fertility tracker	pregnancy/fertility related
10.		SystmOnline - patient health management app	TPP	Makes patient data available, gives patient the ability to request repeat prescriptions and book appointments from the app.	consulting/communication
